# Effects of Supplementation with an Herbal Mixture on the Antioxidant Capacity of Milk

**DOI:** 10.3390/ani13122013

**Published:** 2023-06-16

**Authors:** Magdalena Stobiecka, Jolanta Król, Aneta Brodziak, Renata Klebaniuk, Edyta Kowalczuk-Vasilev

**Affiliations:** 1 Department of Quality Assessment and Processing of Animal Products, Faculty of Animal Sciences and Bioeconomy, University of Life Sciences in Lublin, Akademicka 13, 20-950 Lublin, Poland; magdalena.stobiecka@student.up.edu.pl (M.S.); aneta.brodziak@up.lublin.pl (A.B.); 2 Institute of Animal Nutrition and Bromatology, Faculty of Animal Sciences and Bioeconomy, University of Life Sciences in Lublin, Akademicka 13, 20-950 Lublin, Poland; renata.klebaniuk@up.lublin.pl (R.K.); edyta.kowalczuk@up.lublin.pl (E.K.-V.)

**Keywords:** milk, Holstein-Friesian cows, herbal mixture, supplementation, antioxidant capacity

## Abstract

**Simple Summary:**

The content of antioxidant components and the antioxidant potential of milk can be modified through animal nutrition, i.e., with the inclusion of various natural additives, e.g., herbs, seeds, or byproducts, to the feed ration. The aim of this study was to assess the effect of the addition of a standardized herbal mixture (oregano, common thyme, purple coneflower, and cinnamon bark) to the feed ration for Holstein-Friesian cows on the antioxidant capacity of milk. This study demonstrated the potential of the herbal blend to increase the content of bioactive ingredients with antioxidant properties in milk, i.e., whey proteins (β-lactoglobulin, lactoferrin) and lipophilic vitamins (A, E). The value of the antioxidant potential of milk increased as well; from a nutritional point of view, this seems to be of particular importance for the protection of the organism against the harmful effects of oxidative stress.

**Abstract:**

The aim of this study was to assess the effect of the addition of a standardized herbal mixture to the feed ration for Holstein-Friesian cows on the antioxidant capacity of milk. The study was carried out on a farm specialized in breeding dairy cattle. The exact study involved 30 cows in lactation III, which were in the first phase of lactation at the beginning of the experiment (15 cows—control group; 15 cows—experimental group). The nutrition supplied to the cows was based on the TMR (total mixed ration) system, with roughage and concentrate fodder used as the basis of the feed ration. The addition of a standardized blend of dried herbs, i.e., oregano (*Origanum vulgare*), thyme (*Thymus vulgaris*), purple coneflower (*Echinacea purpurea*), and cinnamon bark (*Cinnamomum zeylanicum*), was the experimental factor. Powdered herbs were administered as a component of the concentrate fodder at the dose of 3% DM ration/day/head. Milk samples were collected four times during the experiment (term 0 after the colostrum period and then after lactation weeks 2, 4, and 6). The following parameters were determined in the milk: the basic chemical composition, i.e., the content of total protein, fat, lactose, and casein; somatic cell count; content of selected whey proteins (α-lactalbumin, β-lactoglobulin, lactoferrin, BSA); and fat-soluble vitamins (A, D_3_, E). Additionally, the milk antioxidant capacity (ABTS, FRAP, DPPH) was determined and the degree of antioxidant protection (DAP) was calculated. It was shown that the milk from cows receiving the herbal blend-supplemented fodder had a higher content of casein, compared to the control group. The herbal supplementation contributed to a significant increase in the content of bioactive compounds, i.e., selected whey proteins (β-lactoglobulin, lactoferrin) and lipophilic vitamins (A, E). The milk was also characterized by significantly higher antioxidant potential (regardless of the measurement method) and a higher degree of antioxidant protection (DAP).

## 1. Introduction

The content of antioxidant components in milk and its antioxidant potential can be modified through animal nutrition, e.g., inclusion of various natural additives to feed rations. The dairy cow nutrition is most often supplemented with herbs. With their high content of biologically active substances, herbs exert a positive effect on the cow organism, which in turn is reflected in the quality of milk [1,2]. Of note, feed additives used in cattle nutrition can also serve protective functions and act as metabolic regulators [3,4]. Consequently, they contribute to an increased resistance of animals exposed to stress (changes in the feed ration, thermal stress), enhance the absorption of essential nutrients, and increase the degree of antioxidant protection [3,5]. This aspect is particularly important in high-yielding animals, which are characterized by reduced immunity and, consequently, increased susceptibility to diseases [6,7]. Pasture grazing significantly increases the content of antioxidant compounds in milk, thus increasing its antioxidant potential [8,9,10,11,12]. Improvement is more difficult to achieve when cows are fed with preserved fodder, especially silage [13,14,15]. As reported by Nielsen et al. [13], a higher proportion of maize silage in cow rations is one of the causes of the lower content of vitamins and antioxidants in milk. In turn, Alves et al. [14] have found that maize silage, which is most often used in cow nutrition, has a low content of carotenoids.

It should be emphasized that herbs are a natural source of antioxidants [16]. These primarily include carotenoids (xanthophylls and carotenes) and polyphenols (flavonoids, anthocyanins, phenolic acids, stilbenes, and lignans), as well as alkaloids, terpenes, saponins, and essential oils [17,18,19]. Already at the stage of storage, they protect fodder against spoilage through inhibition of the oxidation process [20]. Given their antioxidant properties, the following herbs are most often used in animal nutrition: oregano (*Origanum vulgare*), cinnamon (*Cinnamonum cassia*), rosemary (*Salvia rosmarinus*), thyme (*Thymus vulgaris*), turmeric (*Curcuma longa*), cumin (*Carum carvi*), ginger (*Zingiber officinale*), stinging nettle (*Urtica dioica*), and purple coneflower (*Echinacea purpurea*) [2,21,22,23,24]. Even their low concentrations in feed mixtures have an impact on the antioxidant indices in lactating dairy cows. Animals whose diets were supplemented with herbs exhibited an increase in the activation of antioxidant enzymes in both blood and milk, which play an important role in cell protection against oxidative damage [25,26,27]. Many studies have demonstrated that herbs used in the diet for ruminants have a beneficial effect, as they improve the feed conversion efficiency, modify the rumen microflora, and, consequently, improve animal health and performance [27,28,29,30,31,32,33]. Better production results are achieved upon application of herbal blends rather than individual herbs, mainly due to the synergistic effect of their active compounds [34]. Importantly, bioactive plant compounds are highly resistant to microbial degradation in the rumen and do not lose their functionality [35].

Modern consumers are becoming increasingly aware of the important role of antioxidant compounds in supporting and strengthening the defense mechanisms in the organism, which is essential for prevention of such lifestyle diseases as cardiovascular diseases, cancer, diabetes, and obesity. Extremely valuable in this respect are antioxidant compounds derived from natural sources. Similarly, milk is a source of antioxidants. They are mainly contained in the protein fraction (β-lactoglobulin (β-Lg), lactoferrin) and in the fat fraction (vitamins E and A, β-carotene) [36,37,38,39].

To meet consumer expectations, nutritional solutions for full optimization of the production potential of cows and improvement of the composition of their milk are being sought. Therefore, the present research was undertaken to assess the influence of supplementation of feed rations for Holstein-Friesian cows with a dedicated phytobiotic-rich herbal mixture on the level of antioxidant capacity of milk.

## 2. Materials and Methods

### 2.1. Experimental Material

This study was carried out on a farm specialized in breeding dairy cattle of the Holstein-Friesian breed. The experiments were approved by the 2nd Local Ethics Committee for Animal Testing in Lublin (Resolution No 36/2011 of 7 June 2011). During this research, the herd comprised 86 dairy cows with an average milk yield of 7860 kg. The exact study involved 30 cows in lactation III selected from the herd, which were in the first phase of lactation at the beginning of the experiment (15 cows—control group; 15 cows—experimental group) (Table 1). The average body weight of the cows was 655 kg.

The nutrition supplied to the cows was based on the TMR system (Table 2). The basis of the feed ration included roughage (RO): maize silage, grass haylage, beet pulp silage, wheat straw, and concentrate fodder (CF) as required. The experimental factor was the addition of a standardized blend of dried herbs: oregano (*Origanum vulgare*), thyme (*Thymus vulgaris*), purple coneflower (*Echinacea purpurea*), and cinnamon bark (*Cinnamomum Zeylanicum*). The formula of the herbal mixture selected for this study was developed during investigations carried out at the Institute of Animal Nutrition and Bromatology, University of Life Sciences in Lublin. Initially, this research covered several dozen species of herbs, e.g., purple coneflower (*Echinacea purpurea*), oregano (*Origanum vulgare*), thyme (*Thymus vulgaris*), cinnamon (*Cinnamomum*), common garlic (*Allium sativum*), licorice (*Glycyrrhiza glabra*), and caraway (*Carum carvi*). Next, the composition of the mixture was established based on the analyses of the chemical composition and content of biologically active substances in the herbs with consideration of the synergistic and antagonistic effects of compounds contained in the herbal raw materials. In addition to the basic composition, the content of essential oils in the experimental blend was determined with the method of 1-h steam distillation in the Deryng apparatus. The distillation process was carried out with the addition of 400 mL of demineralized water. Samples of essential oils were diluted 100-fold with hexane (10 µL of the sample + 990 µL of the solvent) prior to further analysis. Subsequently, the diluted essential oils were subjected to chromatographic analysis using the GC-MS system (Shimadzu GCMS-TQ8040, Kioto, Japan) in the Scan mode.

Powdered herbs were administered to the cows from the experimental group as a component of the concentrate fodder at the dose of 3% DM ration/day/head. Milk samples were collected 4 times during the experiment (after the colostrum period and then after lactation weeks 2, 4, and 6).

### 2.2. Analysis of the Chemical Composition and Nutritional Value of Fodder

The content of dry matter, total protein, crude fiber and its fractions, crude fat, and crude ash was determined in the feed samples in accordance with the currently applicable standards [40]. The content of nitrogen-free extractives (NFE) was calculated mathematically. Additionally, the content of some plant secondary metabolites was determined in the concentrate fodder and the herb-supplemented feed [41]. The nutritional value of the feed and rations was estimated based on the results of the basic analysis of the feed with the use of the Winwar 1.6 computer program [42], and the nutritional values were expressed in INRA units [43] (Table 3).

### 2.3. Analyses of Milk

The basic chemical composition in each milk sample, i.e., the content of total protein, fat, lactose, and dry matter, was determined using the Infrared Milk Analyzer (Bentley Instruments, Chaska, MN, USA).

#### 2.3.1. Determination of Protein

The content of casein was determined according to AOAC [44]. However, the concentration of selected whey proteins, i.e., α-lactalbumin (α-La), β-lactoglobulin (β-Lg), lactoferrin, and bovine serum albumin (BSA), was determined using reversed-phase high-performance liquid chromatography according to the methodology described by Brodziak et al. [45]. All samples for the whey protein determinations were prepared as in Romero et al. [46] with modifications of Brodziak et al. [45].

#### 2.3.2. Determination of Vitamins

The reversed-phase high-performance liquid chromatography (RP-HPLC) was also used to determine the concentration of fat-soluble vitamins, i.e., A, D_3_, and E. All samples were prepared with the Röse-Gottlieb fat extraction method modified by Hewavitharan et al. [47]. The separations and identifications were conducted according to Brodziak et al. [11].

#### 2.3.3. Determination of Antioxidant Capacity

The antioxidant capacity of the milk was determined with three methods, i.e., FRAP by Benzie and Strain [48], DPPH by Brand-Williams et al. [49], and ABTS by Sahin et al. [50]. The samples for the analysis were extracted from fresh milk, i.e., 10 mL of the solvent was added to 1 mL of milk (1 M HCl solution in 95% ethanol in a volume ratio of 15/85), shaken for 1 h at 40 °C in a rotary shaker at 300 rpm/min, and centrifuged in a centrifuge at 8000× *g* (MPW-350, Med. Instruments, Warsaw, Poland) for 10 min. The extract was used to determine the antioxidant activity.

FRAP Assay. The ferric reducing antioxidant power (FRAP) assay was conducted according to the method developed by Benzie and Strain [48]. The method is based on the reduction of the Fe^3+^-TPTZ (ferric tripyridyl triazine) complex to Fe^2+^-TPTZ at low pH. The reduction is reflected by appearance of the blue color. The absorbance readings were taken using a UV-Vis spectrophotometer (U-2900 HITACHI, Tokyo, Japan) at 593 nm. Different concentrations of Trolox were used as standards for calibration. The results were expressed as milligrams of Trolox equivalents (TE) per 100 mL of sample.

DPPH Assay. The DPPH assay was carried out as described by Brand-Williams et al. [49]. A total of 0.50 mL of the extract was transferred into a test tube, and 3.0 mL of a 6 × 10^−5^ M methanolic DPPH (2,2-diphenyl-1-picrylhydrazyl) solution was added. The absorbance readings were taken using a UV-Vis spectrophotometer (U-2900 HITACHI, Tokyo, Japan) at 515 nm against a reagent blank after being kept in the dark for 30 min. Different concentrations of Trolox were used as standards for calibration. The results were expressed as the amount of DPPH radical-reducing antioxidant compounds contained in a 100 mL sample equivalent to milligrams of TE after 60 min of reaction.

ABTS Assay. The total antioxidant capacity of the samples was determined using the radical cation ABTS^+^ (2,2′-azino-bis(3-ethylbenzothiazoline-6-sulfonic)) decolorization method [50]. The determination method consists in the neutralization of the blue cation radical ABTS, which is manifested with a decrease in the absorbance of the solution. The absorbance was recorded using a UV-Vis Spectrophotometer (Cary 100, Varian, Palo Alto, CA, USA) at 734 nm against a reagent blank. A standard curve was prepared using different concentrations of Trolox, and the results are expressed as milligrams of TE equivalents per 100 mL of sample.

#### 2.3.4. Determination of Cholesterol

The total cholesterol content was determined with the colorimetric enzymatic method with cholesterol esterase and cholesterol oxidase using the Liquick Cor-CHOL kit (Cormay, Łomianki, Poland).

#### 2.3.5. Determination of Degree of Antioxidant Protection

Additionally, the degree of antioxidant protection (DAP) was calculated according to Pizzoferrato et al. [51]. The DAP proposed as the tracing parameter was calculated as the molar ratio between antioxidant compounds and a selected oxidation target [51]:

$$\mathrm{DAP}=\frac{\sum_{n=1}^{n}{\mathrm{AC}}_{1}\left(\mathrm{no}.\mathrm{moles}\right)}{\mathrm{OT}\;\left(\mathrm{no}.\mathrm{moles}\right)}$$

#### 2.3.6. Statistical Analysis

The results were analyzed statistically using a one- and multi-way analysis of variance in StatSoft Inc. Statistica ver. 13 (Dell, Round Rock, TX, USA). Significant differences between the means were determined using Tukey’s test at the significance level *p* (alpha) = 0.05 and 0.01. The results are presented as the means ± standard deviation (SD).

## 3. Results and Discussion

### 3.1. Milk Yield and Basic Physico-Chemical Parameters in Milk

There was no significant effect of the addition of the herbal blend on the milk yield; however, the amount of milk produced by the cows from the experimental group was higher by 2.4 kg per day (Table 4). According to many authors [4,28,52,53], higher milk production after herbal supplementation may be attributed to the galactopoietic effect of the active compounds present in the essential oils. The herbal essential oils can act as galactagogues by enhancing prolactin production and releasing somatotropins, resulting in increased glucose levels in the udder and improved milk production. The milk in the experimental group was characterized by higher levels of the basic components (dry matter, protein, fat, lactose); however, the differences were not statistically significant. For milk yield and protein content, a significant (*p* ≤ 0.05) effect of the week was found. In turn, after supplementation of cow nutrition with the oregano extract (10 g/d), Kolling et al. [54] found no significant effect of this additive on the milk yield and the content of the majority of the milk components. A decrease in the milk fat content was the only effect of the supplementation. In a subsequent study conducted by Kolling et al. [25], extracts of oregano and green tea administered separately or in combination exerted a clear effect on feed intake, milk yield, and antioxidant indices in lactating dairy cows. In turn, Benchaar [55] reported that oregano oil and carvacrol administered at a dose of 50 mg/kg of dry matter in the diet did not have a beneficial effect on rumen fermentation and did not improve nutrient conversion or milk yields. The addition of 10 g of oregano extract per day in cow nutrition resulted in a significant (*p* ≤ 0.05) reduction in SCC [26]. As suggested by the authors, this was associated with the antibacterial properties of oregano; its main components, carvacrol and thymol, have an impact on the properties of the cytoplasmic membrane and can inhibit bacterial adhesion to epithelial cells. In turn, no effect of the plant extracts on the milk yield and basic composition was recorded [26]. Similar conclusions were formulated by Olijhoek et al. [56] in a study on dried oregano supplementation in cow nutrition. Similar to the present study, Węglarzy et al. [57] did not find a significant effect of supplementation with fresh purple coneflower and caraway herbs on the cow milk yield. Nevertheless, the supplementation contributed to a significant increase in the content of protein and fat in the milk. In turn, as reported by Ghafaria et al. [58], the addition of cumin seeds (*Cuminum cyminum*) improved the milk yield, with a slight decrease in the fat content and cholesterol levels. In the present study, the milk from the experimental group was characterized by a lower cholesterol level, but the differences were not significant. A 25% decrease in the level of total cholesterol was found after supplementation of cow feed rations with a mixture of stinging nettle, dandelion, cumin, and chamomile herbs [59]. Cumin supplementation at a dose of 10 g/day in the diet for lactating Mehsana goats improved the milk yield, nutrient digestibility, and feed conversion rates without adverse effects on hematobiochemical parameters [60]. Nurdin et al. [61] showed that herbal supplementation (black cumin, turmeric) resulted in a decrease in the somatic cell count and significantly increased (*p* ≤ 0.01) the milk yield and protein and lactose levels. The addition of lemongrass and peppermint to feed improved the health of cows and increased the production performance of both dairy and beef cattle [62,63]. Similarly, Kraszewski et al. [28] reported a positive effect of administration of herbal blends (chamomile, yarrow, agrimony, stinging nettle, ribwort plantain, St. John’s wort, and lady’s mantle) on cows. The authors noted an increase in the technological parameters of milk and a significant decrease (by 232,000/mL) in the somatic cell count in the experimental group. In turn, significantly (*p* ≤ 0.05) higher milk yields in a group of cows receiving herbal blends (60% rosemary, 18% cinnamon bark, 18% turmeric, and 4% clove buds) compared with the control group were reported by Hashemzadeh-Cigari et al. [4]. The supplementation with the blend reduced the fat content in the milk (*p* < 0.01) but did not have an effect on the levels of other milk components, i.e., protein and lactose. After rosemary extract supplementation, Kong et al. [64] observed a significant increase in the milk yield and lactose content accompanied by a decrease in the somatic cell count. Kuczyńska et al. [65] carried out a study on four certified organic farms and found beneficial effects of herbal blends (oregano, caraway, and rosemary) on the health of cows and improvements in the nutritional quality of milk. Supplementation of water buffalo rations with herbal blends (black pepper ginger, cinnamon, peppermint, ajwain, and garlic; 20 g/day) had no effect on the milk yield [34]. Nevertheless, the blend was shown to have potential to increase the content of milk fat and unsaturated fatty acids.

The milk from the experimental group contained significantly (*p* ≤ 0.05) more casein, a quantitatively dominant cow milk protein with great importance for the dairy industry. The supplementation contributed to an over 5% increase in the level of this protein, i.e., from 2.75 to 2.91%. It should be emphasized that the presence of the herbal mixture in the feed ration of the experimental group of cows did not change the level of protein supplied in the fodders (PDIN and PDIE values in the concentrate fodder and herb-supplemented concentrate fodder were very close—Table 3). The amount of microbial protein produced in the rumen passing to further sections of the gastrointestinal tract (small intestine) is conditioned by the synchronization of the speed of the fermentation process of carbohydrates in the rumen, especially easily digestible ones, with the rate of decomposition of feed protein [53,66]. However, these carbohydrates increase the formation of propionic acid in the rumen, which stimulates the secretion of insulin, which may affect the uptake of amino acids by the mammary gland [67]. It can, therefore, be assumed that the provision of specific compounds contained in herbs affects the change in amino acid protein synthesis in the rumen (synthesis of microorganisms), as well as the availability of specific amino acids in the small intestine for the synthesis of milk proteins.

### 3.2. Bioactive Proteins in Milk

The use of phytobiotic-rich herbs has an impact on the health-promoting value of milk, which is mainly associated with the activity of phenolic compounds, i.e., flavonoids characterized by strong antioxidant and antibacterial properties [17]. The milk samples from the experimental group contained significantly higher amounts of whey proteins (by 0.09 p.p.) than the milk from the control group (Table 5). The main part of whey proteins is constituted by albumins, i.e., α-lactalbumin, β-lactoglobulin, and bovine serum albumin, the so-called blood serum albumin. Higher levels of these proteins were detected in the milk of the experimental group, with significant differences found only in the β-Lg level (*p* ≤ 0.05). It should be emphasized that whey proteins, in particular β-Lg, have the highest antioxidant potential of all proteins in the human diet.

This is associated with the high content of sulfur amino acids, especially cysteine, which is indispensable for the synthesis of glutathione [68,69]. Antioxidant activity is also exhibited by lactoferrin, as it chelates iron, thereby enhancing its bioavailability and inhibiting its pro-oxidative activity. The raw material produced by the cows from the experimental group was a richer source of this protein; its level was on average 30% higher (*p* ≤ 0.01) than in the control group. It also contained a 20% higher amount of lysozymes (*p* ≤ 0.05). Of note, lactoferrins and lysozymes play a significant role in the protection of the organism, as they are one of the most important components of non-specific immune mechanisms [70]. The tendencies observed in the present study are in agreement with the results reported by Kuczyńska et al. [65]. Already after the first week of application of herbal blends (oregano, rosemary, and cumin) to cow feed rations, the levels of whey proteins in milk were found to increase. In a study conducted by Klebaniuk et al. [71], herbal blends (thyme, oregano, cinnamon, and purple coneflower) administered to cows during the dry period exerted a beneficial effect on the quality of colostrum (increased content of immunoglobulins). In turn, Reklewska et al. [72] reported a higher level of lactoferrin in milk after supplementation of feed rations with the purple coneflower (*Echinacea purpurea*) herb. The content of the protein increased significantly (*p* < 0.01) during the administration of the supplement and after cessation of the supplementation (over 14 days). As suggested by the authors, the immunostimulating effect of the purple coneflower herb was associated with the presence of caffeic acid derivatives, which stimulate the activity of immune cells, have antiviral activity, and are strong antioxidants.

### 3.3. Lipophilic Vitamins in Milk

The results shown in Table 6 indicate a significant effect of the supplementation with the herbal blend on the content of lipophilic vitamins in milk. A higher level of these vitamins was found in the milk of the experimental group, with significant differences noted in the case of vitamin A (*p* ≤ 0.05) and E (*p* ≤ 0.01). In comparison with the control group, the milk from the experimental group contained approximately 30% and 40% higher contents of vitamins A and E, respectively. It should be emphasized that fat-soluble vitamins, primarily E and A, are the main antioxidants derived from the fat fraction protecting cells against reactive oxygen species [36,39]. Their activity consists of scavenging organic free radicals and inhibiting lipid peroxidation [73]. Similarly, Kuczyńska et al. [65] reported an increase in the content of vitamins E and A in milk fat after supplementation with a mixture of oregano, cumin, and rosemary. The concentration of vitamin E was found to increase on average by 50% after the 21-day supplementation period. These changes are explained by the authors by the content of phytobiotics and better utilization of dietary ingredients. Changes in the content of vitamins in milk found in our own research may also be a consequence of changes taking place in the rumen of ruminants, as all cows received the same mineral–vitamin additives. The observed phenomenon is a complex issue, simultaneously mobilizing for further research. Leiber et al. [74], however, showed that cows grazing on alpine pastures produced milk containing 86% higher levels of vitamin E than milk from cows fed with preserved fodder.

### 3.4. Antioxidant Capacity of Milk

**Figure 1:**
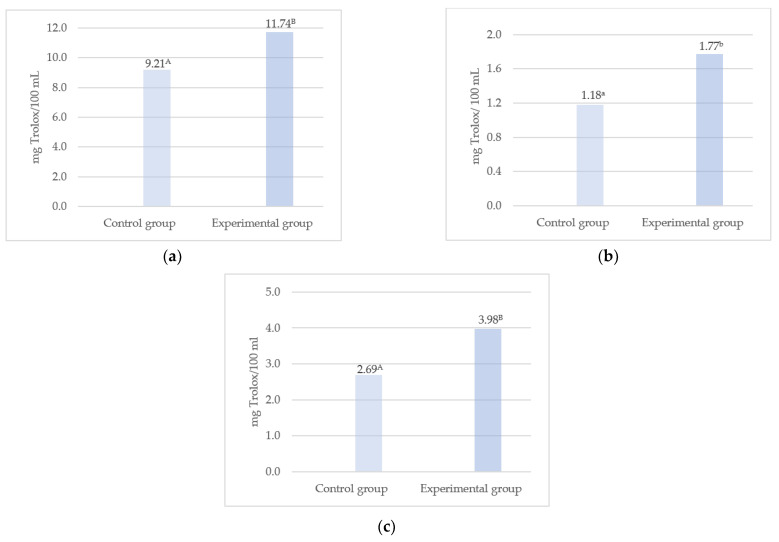
Antioxidant activity of milk expressed in mg of Trolox equivalent per 100 mL of sample. (**a**) FRAP—ferric reducing antioxidant power assay, (**b**) DPPH—2,2-diphenyl-1-picrylhydrazyl assay, (**c**) ABTS—2,2′-azino-bis(3-ethylbenzothiazoline-6-sulfonic acid) assay. ^a, b^—significant differences at *p* ≤ 0.05; ^A, B^—significant differences at *p* ≤ 0.01.

The modification of the cow’s diet significantly increased the antioxidant potential of milk, regardless of the determination method used (FRAP, DPPH, ABTS). The raw material obtained from the experimental group of cows was characterized by significantly higher antioxidant potential than the control (Figure 1a–c). In comparison with the control, the DPPH and ABTS values increased by approximately 50%. In the FRAP assay, the milk exhibited approximately 20% higher Fe^2+^ chelation capacity. The higher antioxidant activity of these milk samples relative to the control was associated with the introduction of natural antioxidants, i.e., phenolic compounds, together with the herbal blend. Plant extracts may contribute to an increase in endogenous antioxidants and free radical scavenging [35]. In a study conducted by Paraskevakis [75], oregano supplementation of the diet for goats induced a significant (*p* < 0.001) increase in the antioxidant value of milk (expressed as FRAP). In another study by Uegaki et al. [76], the diet for Holstein cows was supplemented with three herbs, i.e., lemongrass, peppermint, and basil, for 14 days. A significant increase in the antioxidant activity of milk, compared to the control, was noted in all variants. As suggested by Yilmaz-Ersan et al. [69], the differences in the total antioxidant potential of milk can be explained by the differences in its chemical composition. Of note, the milk from the experimental group analyzed in the present study contained a higher level of antioxidants, hence the increase in its antioxidant potential. An increase in the TAS level following supplementation with herbs was reported by Kuczyńska et al. [65]. The antioxidant potential of milk from cows receiving phyto-additives increased significantly. An almost three-fold increase in the level of TAS, which indicates an increase in the degree of antioxidant protection, was detected. In a study conducted by Qingru et al. [77], the introduction of a Chinese herbal formula in cow nutrition resulted in an over 40% (*p* < 0.01) increase in the total antioxidant capacity of milk and an over 20% (*p* < 0.01) decline in the malondialdehyde (MDA) levels. As demonstrated by Zhang and Zhao [78], 400 and 600 mg/kg doses of the Chinese herb extract supplemented in cow nutrition may have a beneficial effect on milk production through improvement of animal health and enhancement of antioxidant activity. Various studies indicate that pasture grazing as part of cow nutrition has a significant impact on the content of lipophilic antioxidants in milk and, consequently, its antioxidant activity [39,79]. Santa et al. [80] reported that milk from grazing Jersey cows versus those fed TMR diets contained significantly higher levels of α-tocopherol (0.74 vs. 0.27 mg/100 g), retinol (125.62 vs. 57.51 µg/100 g), and β-carotene (0.69 vs. 0.41 µg/100 g), which was reflected in the higher antioxidant activity of the milk (3.02 vs. 2.53 µmol TE/mL).

### 3.5. Degree of Antioxidant Protection of Milk

**Figure 2:**
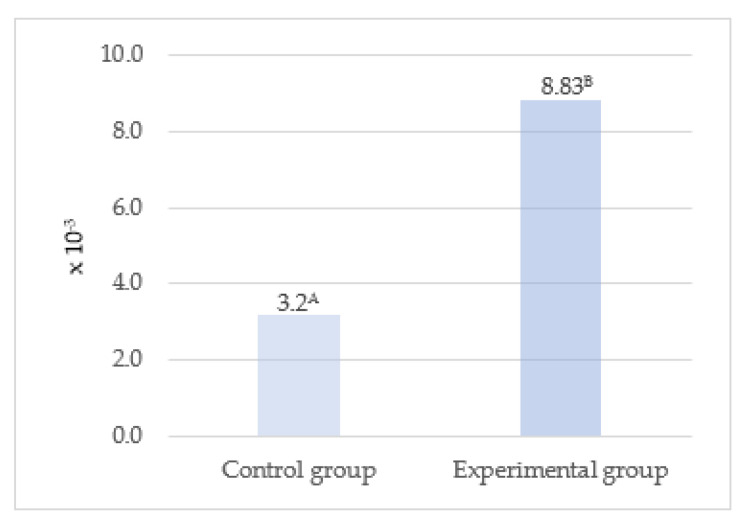
Degree of antioxidant protection of milk. ^A, B^—significant differences at *p* ≤ 0.01.

For determination of product quality, Pizzoferrato et al. [51] proposed that the degree of antioxidant protection (DAP) should be calculated as a molar ratio between antioxidant compounds and oxidants. In milk, vitamins E and A are the antioxidant compounds and cholesterol is the target molecule for oxidation. Products of cholesterol oxidation exert an adverse effect on human health, as they can be absorbed in the gastrointestinal tract into the bloodstream, thus increasing the likelihood of the development of cardiovascular diseases and atherosclerosis [81,82]. It should be emphasized that the negative impact of cholesterol depends on the degree of antioxidant protection of a given product [83]. The higher the DAP value, the higher the oxidation stability of the product. The milk of cows receiving the herbal blend-supplemented fodder exhibited a significantly higher degree of antioxidant protection (DAP) (Figure 2). According to Pizzoferrato et al. [51], milk from pasture-grazed animals has a higher degree of antioxidant protection than milk produced by non-grazed animals, which indicates that green fodder is a rich source of antioxidants, protecting cholesterol against oxidative reactions. As reported by Puppel et al. [12], the highest values of the degree of antioxidant protection (DAP) and total antioxidant status (TAS) were determined in a system with pasture grazing as the basis of cow nutrition. Nevertheless, the addition of maize grain silage was shown to increase the degree of antioxidant protection through an increase in the content of vitamin E in milk. As suggested by the authors, DAP and TAS should be considered as biomarkers of antioxidant changes in milk. Higher levels of both antioxidant protection and total antioxidant potential contribute to higher product stability and quality.

## 4. Conclusions

The herbal mixture supplementation of cow nutrition had no significant effect on the yield and basic chemical composition of the milk. Of note, significant differences in the content of casein, a protein of high importance for the dairy industry, were observed in favor of the experimental group. The blend was shown to increase the content of bioactive ingredients with antioxidant properties in the milk. The supplementation resulted in a significant increase in the content of selected whey proteins (β-lactoglobulin, lactoferrin) and lipophilic vitamins (A, E). The antioxidant capacity of milk increased as well; from a nutritional point of view, this seems to be especially important for the protection of the organism against harmful effects of oxidative stress. Importantly, the milk of cows receiving the herbal blend-supplemented fodder exhibited a significantly higher degree of antioxidant protection (DAP).

## Figures and Tables

**Table 1:** Experimental design.

Experimental Design	Group	
	C	E
6 weeks	RO + CF	RO + CF + HM (3%)
number of cows	15	15

C—control group cows receiving a standard ration based on roughage used on the farm with the addition of a mixture of concentrate fodder. E—experimental cows receiving a standard ration based on roughage with the addition of a mixture of concentrate fodder and 3% of the experimental herbal mixture (HM). RO—roughage. CF—concentrate fodder mixture: triticale—35%, oats—20%, barley—18%, maize grain—14%, post-extraction rapeseed meal—2.5%, commercial supplementary mixture—10%, fodder chalk and mineral–vitamin additives—0.5%. HM—standardized herbal mixture: thyme 25%, purple coneflower 35%, oregano 25%, and cinnamon 15%.

**Table 2:** Composition of TMR administered to the experimental cows.

Item	Beet Pulp Silage	Maize Silage	Haylage	Wheat Straw	Concentrate Mixture	Herb-Supplemented Concentrate Mixture	Total
Control TMR							
kg DM/d/head	1.60	7.39	3.65	0.44	4.73	-	17.81
Share (%)	9.0	41.5	20.5	2.5	26.5	-	100
Experimental TMR							
kg DM/d/head	1.60	7.39	3.65	0.44	-	4.95	18.03
Share (%)	8.9	41.0	20.2	2.4		27.5	100

**Table 3:** Chemical composition and nutritional value of basic fodders.

Parameter	Type of Feed					
	Beet Pulp Silage	Maize Silage	Haylage	Wheat Straw	Concentrate Mixture	Herb-Supplemented Concentrate Mixture
Dry matter (%)	21.3	26.4	30.4	87.5	87.6	88.4
Content in 1 kg dry matter (g)						
Crude protein	112.1	80.9	161.0	35.0	137.1	136.8
Crude fat	8.8	27.1	33.5	15.5	23.4	23.0
Crude fiber	178.4	200.8	231.7	397.0	41.3	41.9
Ash	70.5	38.1	59.2	42.0	34.5	35.2
NFE	630.2	653.1	514.6	510.5	763.7	763.1
Content of biologically active component (%)						
Linalool	-	-	-	-	-	8.32
Cymene	-	-	-	-	-	7.02
Thymol	-	-	-	-	-	5.83
Carvone	-	-	-	-	-	2.31
Carvacrol	-	-	-	-	-	1.13
Nutritive value of 1 kg DM						
UFL	1.01	0.90	0.80	0.30	1.19	1.19
PDIN	60.0	78.0	88.0	30.0	89.9	89.9
PDIE	84.0	73.0	66.0	40.0	91.9	91.7
LFU	1.10	1.24	1.20	1.30	-	-

NFE—nitrogen-free extractives. UFL—unit for lactation. PDIE—the sum of microbial protein that could be synthesized in the rumen from available energy and the dietary protein undegraded in the rumen but truly digestible in small intestine. PDIN—the sum of microbial protein that could be synthesized in the rumen from available N and the dietary protein undegraded in the rumen but truly digestible in small intestine. LFU—fill unit for lactating cows.

**Table 4:** Yield and basic chemical composition of milk.

Parameter	Control Group	Experimental Group	*p*-Value	
			Treatment	Week
Milk yield (kg)	26.8 ± 2.9	29.2 ± 5.1	ns	0.039
Dry matter (%)	13.09 ± 0.20	13.19 ± 0.44	ns	ns
Protein (%)	3.47 ± 0.52	3.51 ± 0.62	ns	0.042
Casein (%)	2.75 ^a^ ± 0.41	2.91 ^b^ ± 0.54	0.048	ns
Fat (%)	4.27 ± 0.89	4.30 ± 0.87	ns	ns
Lactose (%)	4.65 ± 0.17	4.72 ± 0.13	ns	ns
Cholesterol (mg/L)	369.34 ± 34.76	293.58 ± 55.34	ns	ns
SCC (thousand/mL)	178 ± 220	172 ± 180	ns	ns

^a, b^—significant differences at *p* ≤ 0.05. ns—not statistically significant.

**Table 5:** Content of selected bioactive proteins in milk.

Parameter	Control Group	Experimental Group	*p*-Value	
			Treatment	Week
Whey proteins (%)	0.64 ^a^ ± 0.021	0.73 ^b^ ± 0.064	0.023	ns
β-lactoglobulin (g/L)	2.99 ^a^ ± 0.20	3.14 ^b^ ± 0.23	0.032	ns
α-lactalbumin (g/L)	0.95 ± 0.12	1.03 ± 0.11	ns	ns
Bovine serum albumin (g/L)	0.33 ± 0.06	0.36 ± 0.09	ns	ns
Lactoferrin (mg/L)	103.77 ^A^ ± 8.36	136.19 ^B^ ± 11.19	0.008	ns
Lysozyme (µg/L)	5.62 ^a^ ± 0.86	6.92 ^b^ ± 0.99	0.021	ns

^a, b^—significant differences at *p* ≤ 0.05; ^A, B^—significant differences at *p* ≤ 0.01. ns—not statistically significant.

**Table 6:** Content of selected bioactive proteins in milk.

Parameter	Control Group	Experimental Group	*p*-Value	
			Treatment	Week
Vitamin A (mg/L)	0.354 ^a^ ± 0.004	0.503 ^b^ ± 0.005	0.018	ns
Vitamin E (mg/L)	1.276 ^A^ ± 0.38	2.06 ^B^ ± 0.28	0.006	ns
Vitamin D_3_ (µg/L)	0.66 ± 0.16	0.75 ± 0.14	ns	ns

^a, b^—significant differences at *p* ≤ 0.05; ^A, B^—significant differences at *p* ≤ 0.01. ns—not statistically significant.

## Data Availability

Not applicable.
